# Supplementary material for: Deletion of wfs1 Impairs Oligodendrocyte Precursor Cells Dorsal Distribution and Myelination Through the wfs1-hmgcs1 Axis in Zebrafish
Source: Biology (Basel). 2026 Aug 16;15(16):1402. doi: 10.3390/biology15161402 (PMC13510151; doi:10.3390/biology15161402)
Supplement: Supplementary file 1 [file biology-15-01402-s001.zip › File S1-Transcriptome sequencing data and differentially expressed genes (DEGs) in wfs1-- zebrafish at 5 dpf/2.KEGG/wfs1KOvsWT/ALL/src/dre04920.html]

dre04920


- dre:406473

- Up regulated genes

406473(3.55746093515464)

- dre:100037357

- Down regulated genes

100037357(-1.39102944946719)

- dre:100149042

- Up regulated genes

100149042(3.22834363695363)

- dre:564295

- Down regulated genes

564295\_2(-1.27307321576804)

Close
